# New approach into human health risk assessment associated with heavy metals in surface water and groundwater using Monte Carlo Method

**DOI:** 10.1038/s41598-023-50000-y

**Published:** 2024-01-10

**Authors:** Mohamed Hamdy Eid, Mustafa Eissa, Essam A. Mohamed, Hatem Saad Ramadan, Madarász Tamás, Attila Kovács, Péter Szűcs

**Affiliations:** 1 Institute of Environmental Management, Faculty of Earth Science, University of Miskolc, Miskolc-Egyetemváros, 3515 Hungary; 2 Geology Department, Faculty of Science, Beni-Suef University, Beni-Suef, 65211 Egypt; 3 Division of Water Resources and Arid Land, Department of Hydrogeochemistry, Desert Research Center, Cairo, Egypt; 4 Faculty of Earth Science, Beni-Suef University, Beni-Suef, 62511 Egypt

**Keywords:** Geochemistry, Environmental sciences, Environmental social sciences, Diseases, Risk factors, Health care, Public health

## Abstract

This study assessed the environmental and health risks associated with heavy metals in the water resources of Egypt's northwestern desert. The current approaches included the Spearman correlation matrix, principal component analysis, and cluster analysis to identify pollution sources and quality-controlling factors. Various indices (HPI, MI, HQ, HI, and CR) were applied to evaluate environmental and human health risks. Additionally, the Monte Carlo method was employed for probabilistic carcinogenic and non-carcinogenic risk assessment via oral and dermal exposure routes in adults and children. Notably, all water resources exhibited high pollution risks with HPI and MI values exceeding permissible limits (HPI > 100 and MI > 6), respectively. Furthermore, HI oral values indicated significant non-carcinogenic risks to both adults and children, while dermal contact posed a high risk to 19.4% of samples for adults and 77.6% of samples for children (HI > 1). Most water samples exhibited CR values exceeding 1 × 10^–4^ for Cd, Cr, and Pb, suggesting vulnerability to carcinogenic effects in both age groups. Monte Carlo simulations reinforced these findings, indicating a significant carcinogenic impact on children and adults. Consequently, comprehensive water treatment measures are urgently needed to mitigate carcinogenic and non-carcinogenic health risks in Siwa Oasis.

## Introduction

In developing countries, rapid industrial growth, economic expansion, and urbanization contribute significantly to increased environmental pollution, posing a national and international concern^1–3^. Activities from industries like petrochemicals and heavy automotive production release pollutants, including toxic metals, organic pollutants, pesticides, microplastics, and emerging pollutants, threatening groundwater resources and human health, environmental services, and sustainable development^4^. Recent studies worldwide report groundwater contamination with lead, iron, manganese, cadmium, copper, and chromium^4^. Cadmium contamination, a global issue mainly affecting Asia and Africa, poses risks to food and water supplies. Cd, even at low concentrations, is highly toxic, leaching into the soil and bio-accumulating in ecosystems^5^.

The global concern regarding the presence of heavy metals in the environment has significantly grown due to their potential negative impacts on human health. These metals are well-known toxins that can cause organ damage and exhibit teratogenic and carcinogenic effects^6,7^. Groundwater contamination with heavy metals has been linked to severe health implications, including kidney damage, degenerative neurological conditions, respiratory and cardiovascular diseases, and cancer^8,9^. Because of their persistent nature, potential toxic elements (PTEs) tend to accumulate in groundwater, constituting a primary route of exposure for humans^10^. Given these severe health risks, public authorities regularly monitor the concentration of various PTEs to mitigate potential hazards to public health. Building upon this backdrop of groundwater's significance and the challenges it confronts, the current study delves into the environmental and human health risks associated with heavy metal contamination in Siwa Oasis.

While essential for metabolism, copper (Cu), zinc (Zn), iron (Fe), and manganese (Mn) can become hazardous when their levels in drinking water exceed permissible limits. Heavy metals can enter the human body through various pathways, including oral consumption, dermal contact, and inhalation^11–13^. These contaminants can be found in drinking water sources, such as surface water and groundwater^14,15^, vegetables, and air^16^. Metals in the environment can be attributed to industrial, agricultural, domestic, medical, and technological activities. When the levels of heavy metals in drinking water exceed the limits set by international organizations, it can lead to various health problems^8^. Ensuring the protection of the environment and human health is crucial, and this involves assessing water quality. The first step in this process is evaluating water quality and identifying pollutant sources to mitigate pollution levels. Effective methods for evaluating the environmental and human health risks associated with heavy metals include the heavy metal pollution index (HPI), metal index (MI), hazard quotient (HQ), hazard index (HI), and carcinogenic risk (CR), integrated with Monte Carlo simulation^2–4,9,16–20^. Furthermore, cluster analysis and principal component analysis (PCA) are valuable tools for classifying the sources of heavy metals and understanding hydrochemical processes in surface water and groundwater^14,21–23^. Contaminated water resources pose severe threats to humans and animals, giving rise to major biological and chemical concerns. Groundwater resources worldwide are increasingly affected by depletion and pollution, and this issue extends to Egyptian deserts, where Siwa Oasis heavily relies on groundwater for drinking and irrigation^24^. Statistics reveal that over one billion people lack access to safe drinking and irrigation water, resulting in approximately 25,000 annual deaths in developing countries^25^.

Siwa Oasis, located in Egypt's northwestern desert, mainly relies on groundwater for drinking and irrigation^24^. Groundwater sources within the Oasis Oasis include groundwater from aquifers like the Nubian sandstone aquifer and shallow aquifers like the Tertiary carbonate aquifer. The groundwater supports agricultural activities and domestic use in Siwa Oasis^26^. Salt lakes within the OasisOasis serve as outlets for water originating from cultivated lands, natural springs, and artesian wells. The growing demand for groundwater in Siwa Oasis, driven by population growth, agriculture, and tourism, has led to the establishment of numerous wells. However, the random placement of these wells and excessive extraction may lead to decreased water pressure and quality. Unauthorized drilling further exacerbates these issues, emphasizing the need for comprehensive studies to monitor the quantity and quality of this limited water resource^27^.

This study aims to comprehensively investigate the environmental and human health risks associated with eight heavy metals in various water resources of Siwa Oasis. The objectives are: (1) Recognize the potential sources using the Spearman correlation matrix, principal component analysis, cluster analysis, and kriging interpolation. (2) Determine the geochemical processes controlling the water chemistry. (3) Applying an innovative approach through integrating several water quality indices (HPI, MI, HQ, HI, and CR) with deterministic and probabilistic (Monte Carlo simulation) methods to assess carcinogenic and non-carcinogenic health risks associated with heavy metal contamination in Siwa Oasis. (4) Using Python programming code to facilitate Monte Carlo simulations, offering precision and efficiency. Extensive libraries and statistical capabilities enable the handling of uncertainty and variability in input parameters, giving more accurate risk estimations. This integration denotes a significant improvement in the assessment of heavy metal pollution.

## Materials and methods

### Study area description

**Figure 1:**
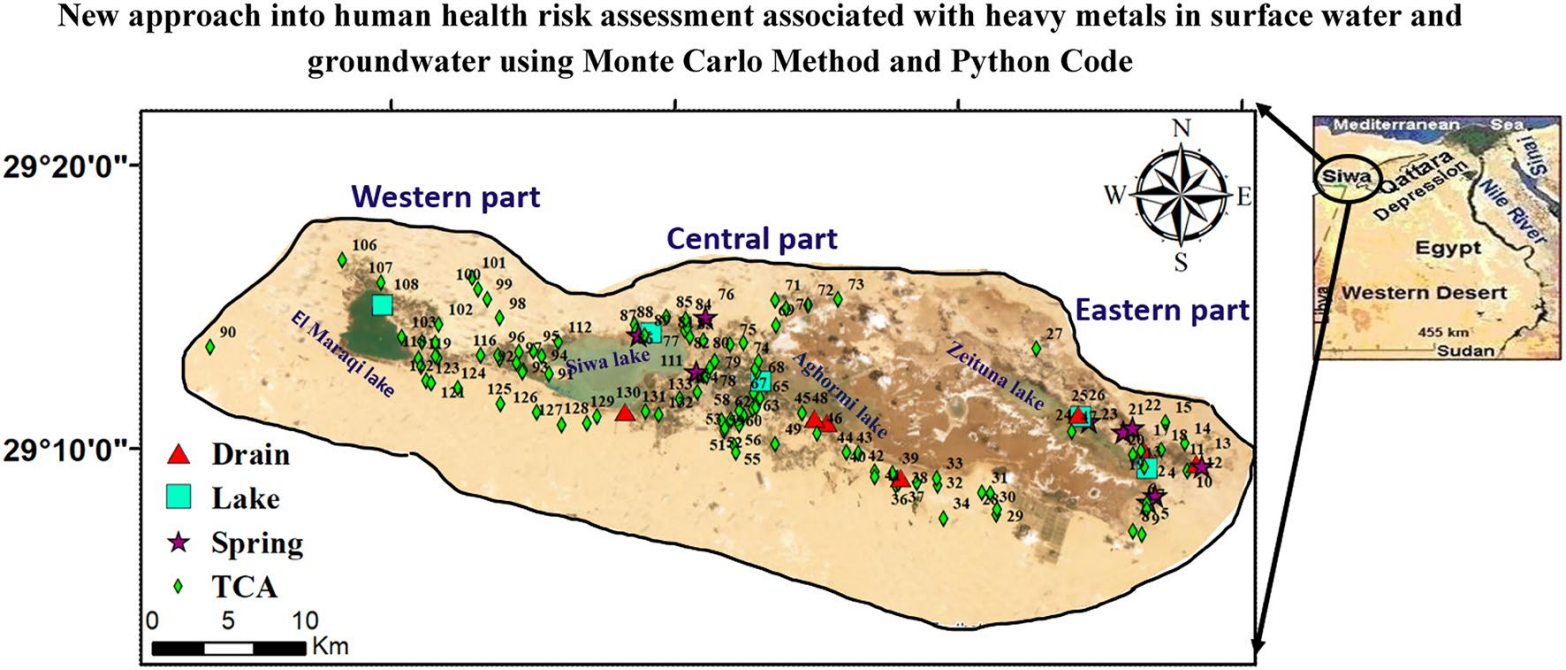
Location and sampling map of Siwa depression.

Siwa Oasis, located in the western desert of Egypt, is a depression that the Mediterranean Sea surrounds to the north, the Libya-Egypt border to the west, and Cairo to the east (Fig. 1). It is positioned at latitude 29°12' N and longitude 25°43' E. The main economic activities in this OasisOasis include agriculture, with palm trees, olives, and various fruits and vegetables being cultivated. Industrial pursuits such as bottling mineral water and extracting olive oil are also prominent^28^. Siwa Oasis covers a land area of 1100 square kilometers and had a population of around 23,546 as of 2010. The climate in Siwa Oasis is characterized by dryness with a high evaporation rate of 16.8 mm per day, which decreases to around 5.4 mm per day during winter. Precipitation in the area is minimal, with a rainfall of about 10 mm^29^. This arid climate and its isolation and limited water resources present unique challenges and opportunities for residents and industries in Siwa Oasis^30^.

### Geology and water resources of Siwa depression

**Figure 2:**
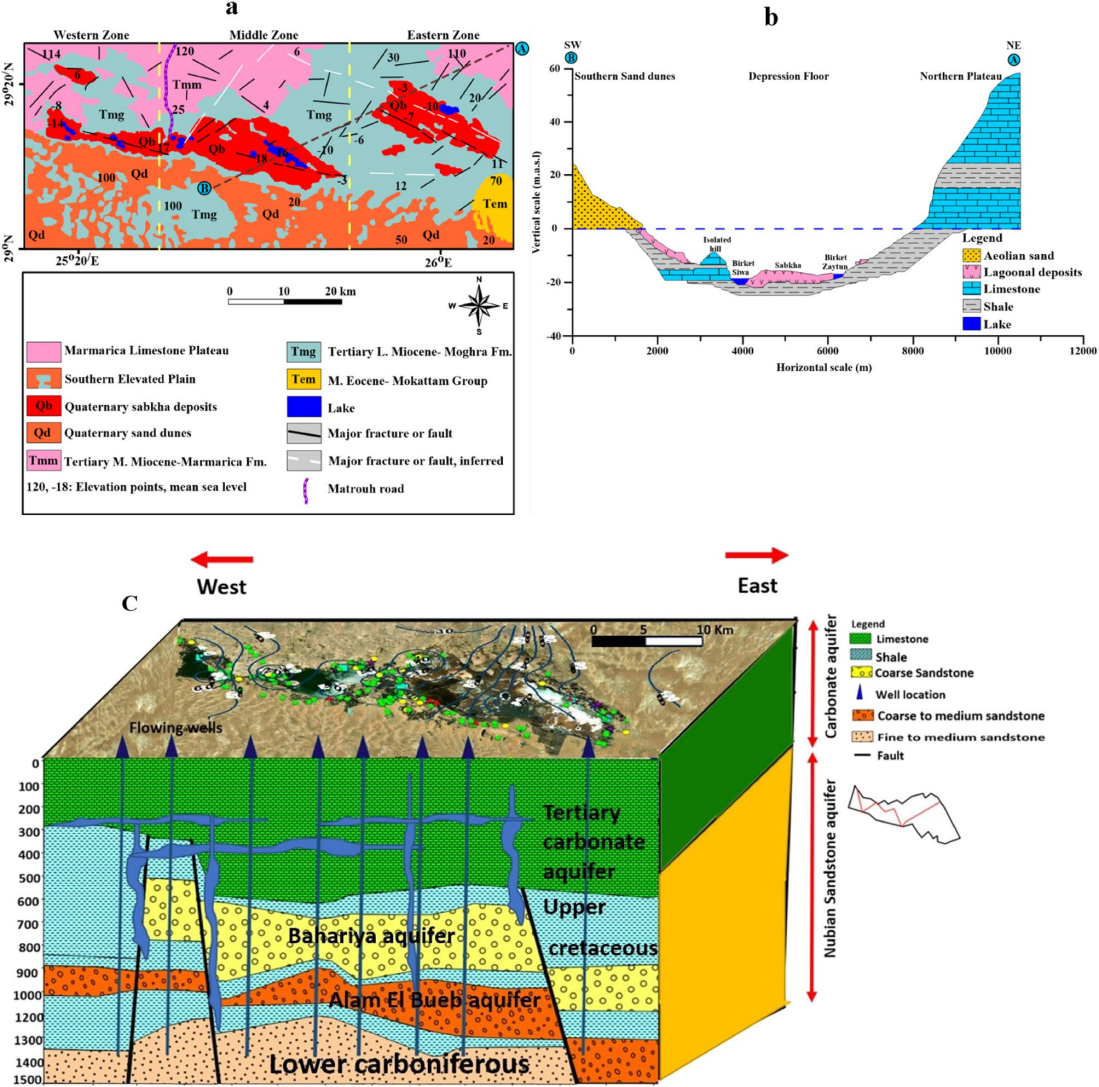
Surface geological map modified after^33^ (**a**), geomorphological map modified after^33^ (**b**), hydrogeological conceptual model and subsurface geological formations in Siwa Oasis (**c**).

The Siwa Oasis has a landscape with different layers of rocks. These include deposits like dunes and salt flats and older layers from the Middle Eocene period made up of limestone and shale (Fig. 2a,b). There are also layers from different geological eras, such as the Palaeozoic, Mesozoic, and Cainozoic^31^. Regarding water supply, Siwa has five aquifers ranging from ones in deposits to a deeper one called the Nubian sandstone aquifer (Fig. 2c). The primary irrigation and domestic use source come from the Miocene aquifer (Tertiary carbonate aquifer), while NSSA (deep aquifer) is mainly used for drinking. However, there are challenges in this region, including soil salinization and waterlogging that mainly occur near salt lakes like Zeitoun, Aghormi, Siwa, and Maraqi. Although these lakes receive water from the groundwater, they have salinity levels, which makes their water unsuitable for domestic or aquatic purposes. Proper management of water resources is crucial to address these issues and ensure the use of the oasis water reserves^32^.

### Sampling and analysis of physicochemical parameters and heavy metals

In February 2022, a field trip was conducted where 133 water samples were collected, including 113 samples from the Tertiary carbonate aquifer (TCA), eight samples from springs, and 12 samples from lakes and drains in polyethylene bottles. These samples were then analyzed chemically at the Desert Research Center in Egypt and Miskolc University in Hungary. During the fieldwork, the pH and electrical conductivity (EC) were measured using portable meters with daily calibration. pH measurements were taken with a WTW model LF 538 pH meter. Electrical conductivity was measured using a YSI model 35 conductivity meter. The methods of Rainwater Thatcher and Friedman were employed. The alkali metal ions (Na^+^ and K^+^) were measured using flame photometry using a standard curve. The hardness (TH) was determined through EDTA procedures, while CO_3_^2−^ and HCO_3_^−^ were analyzed volumetrically. By considering TH and Ca^2+^ contents, the concentration of Mg^2+^ was calculated. To estimate chloride levels accurately, AgNO_3_ titration was employed. To uphold precision, every sample underwent duplicate analysis. In cases where the discrepancy between the total cations and anions surpassed 5%, the sample analysis was reiterated until a satisfactory percentage difference was achieved. The precision and dependability of the results were assured through the utilization of flame photometry and spectrophotometry methods. All chemical data were expressed in mg/L units with a measurement precision assessed through the ionic balance error (IBE) within ± 5%. The total dissolved solids (TDS) were calculated by adding the ions. The ionic balance (IB) was then determined by comparing the percentages of cations and anions with a range, for IB being within ± 5%. Lastly, ICP was utilized to measure the concentrations of heavy metals. Software including surfer 16.6.484 and ArcGIS Pro 2.8.8 were used to create the distribution maps of the sampling location and investigated parameters.

### Cluster analysis (CA)

Cluster Analysis (CA) is a technique used for recognizing patterns in datasets from various sources without supervision. CA identifies features that differentiate groups within the dataset and clusters them accordingly. Both R mode (Row mode) and Q mode (Column mode) approaches have been employed to execute and construct CA. These approaches help create clusters of water samples with characteristics enabling the identification of spatial similarities and grouping of sampling stations based on their hydrogeochemical properties^34^. CA is a tool used in categorizing processes in groundwater (GW), particularly in hydrochemistry investigations, as it plays a crucial role in grouping collected water samples into meaningful geological and hydrogeological categories. A cluster dendrogram is often utilized to represent the clustering process and simplify the complexity of the data, providing a clear depiction of groupings and their relationships^34^.

### Principal component analysis (PCA)

Principal Component Analysis (PCA) is a technique that handles complex multivariate datasets in a linear structure. Its purpose is to analyze data without losing any information while summarizing the dataset and estimating the number of variables needed to explain the variance^33^. By reducing the dimensionality of the data, PCA uncovers hidden patterns and relationships between variables that may not be immediately apparent. To assess groundwater (GW) contamination, the Kaiser Criterion uses eigenvalues from the scree plot to extract principal components. Additionally, the suitability of data for factor analysis is evaluated through tests like Kaiser Meyer Olkin (KMO) and Bartlett's tests, which indicate whether variables are adequate or inadequate within the model. KMO values falling within the ranges of 0.8 to 1, 0.5 to 0.8, and less than 0.5 indicate sufficient, reasonably adequate, and undesirable (or inadequate) data appropriateness, respectively. This comprehensive approach helps researchers gain valuable insights from complex datasets while ensuring that the data adequately represent the underlying relationships^36^.

### Heavy metal pollution index (HPI) and metal index (MI)

The Heavy Metal Pollution Index (HPI) is a tool applied for evaluating the contamination levels of water resources by heavy metals^37^. This index is especially effective in determining if the quality of water is suitable for consumption, considering the presence of heavy metals. The HPI is determined through a method that involves assigning ratings to parameters related to pollution and then calculating a weighted mean using those ratings. Each pollution parameter is given a weight in this process. The rating system usually ranges from 0 to 1. It is determined by considering the importance of each quality factor or by comparing values with recommended reference standards^38^. Specific equations are used to calculate the HPI, as described in Eqs. 1 and 2.

1 $${\text{HPI}}=\frac{{\sum }_{{\text{i}}=1}^{{\text{n}}}{{\text{W}}}_{{\text{i}}}{{\text{Q}}}_{{\text{i}}}}{\sum_{{\text{i}}=1}^{{\text{n}}}{\text{Wi}}}$$

where Q_i_ stands for the sub-index parameter; n is the number of parameters taken for analysis; w_i_ depicts the weight of each parameter, evaluated as 1/S_**i**_; S_i_ symbolizes the standard value of each parameter; Q_i_ represents the sub-index of the boundary, determined by Eq. 2.

2 $${Q}_{i}={\sum }_{i=1}^{n}100 \times \frac{{C}_{i}}{{S}_{i}}$$

The HPI calculation is based on the levels of eight metals: chromium (Cr), copper (Cu), iron (Fe), manganese (Mn), nickel (Ni), lead (Pb), zinc (Zn), and cadmium (Cd). A modified scale with three categories is commonly used to provide an understanding of heavy metal pollution. These categories are classified as excellent water quality when HPI is below 25, good with HPI ranging from 26 to 50, poor quality with an HPI value between 51 to 75, very poor with HPI ranging from 76 to 100, and high pollution risk (unsuitable) when HPI exceeds 100^39^.

On the other hand, the Metal Index (MI) for drinking water offers an assessment of the overall quality of drinking water by considering the potential impact of heavy metals on human health. The MI assumes that the toxicity levels of these metals have a correlation with their concentration, which means they can potentially cause chronic toxic effects on various organs in the human body. The calculation of MI involves an evaluation of the condition where the drinking water quality is compromised if the concentration of a metal exceeds its designated Upper Allowable Limit (UAL) value 38. The following equation (Eq. 3) illustrates the heavy metal index.

3 $${M}_{i}={\sum }_{i=1}^{i}\frac{{C}_{ave}}{{UAL}_{i}}$$

where: C_ave_ signifies the average concentration of each studied HM; UALi stands for the upper allowable limit of the ith metal in the sample. Metal index (MI) has six classes: very clean (MI < 0.3); clean (0.3 < MI < 1); partly affected (1 < MI < 2); moderately affected (2 < MI < 4); heavily affected (4 < MI < 6); and severally affected (MI > 6)^41^.

### Human health risk assessment

The assessment of health risks follows a model recommended by the United States Environmental Protection Agency^42^. Health risk assessment is an analysis examining how environmental pollutants impact health. These risks can be divided into two categories: Carcinogenic (CR) and non-carcinogenic (NCR)^43^. Carcinogenic risks assess the likelihood of developing cancer as a result of prolonged exposure to a pollutant or a combination of contaminants. In contrast, non-carcinogenic risks primarily deal with exposure and include genetic and teratogenic effects^44^. Heavy metals (HMs) found in drinking water sources primarily enter the body through consumption and contact with the skin. Therefore, this study conducts health risk assessments from direct drinking and skin contact, which can be expressed in Eqs. 5 and 6:

5 $${{\text{CDI}}}_{{\text{oral}}}=\frac{{{\text{C}}}_{{\text{w}}}\times {\text{IR}}\times {\text{EF}}}{{\text{BW}}\times {\text{AT}}} \times {\text{ED}}$$

6 $${{\text{CDI}}}_{{\text{dermal}}}=\frac{{{\text{C}}}_{{\text{ave}}}\times {\text{ET}}\times {\text{EF}}\times {\text{Kp}}\times {\text{SA}}\times {\text{CF}}}{{\text{BW}}\times {\text{AT}}} \times {\text{ED}}$$

where CDI _oral_ is the average daily direct intake dose, CDI _Dermal_ is the average daily dose absorbed by the skin, C_w_ is the contents of HMs in the water sample (mg/L), IR is the daily ingestion rate (L/d), EF is the exposure frequency, ED is the exposure duration, BW is the body weight, SA is the exposed skin area, Kp is the skin permeability coefficient, CF is the conversion factor and ET is the exposure time. These exposure parameters are presented in Table 1.

**Table 1:** The parameters for the calculation of HQ, HI, and CR.

HM	Cd	Cr	Cu	Fe	Mn	Ni	Pb	Zn	References
RfD Oral(mg/kg/day)	0.0005	0.003	0.04	0.7	0.024	0.02	0.0014	0.3	^45^
ABS	0.05	0.025	0.3	0.2	0.04	0.04	0.3	0.2	^46^
Rfd Dermal (mg/kg/day)	0.000025	0.000075	0.012	0.14	0.00096	0.0008	0.00042	0.06	^47^
CSF oral mg/kg/day	6.1	0.5					0.5		^48^
CSF dermal	6100	500					500		^48^
Kp	0.001	0.002	0.001	0.001	0.001	0.0002	0.0001	0.0006	^49^
Si	0.003	0.05	3	0.3	0.05	0.07	0.01	1	^50^
ET Adult (h/day)				0.58					^51^
ET Child (h/day)				1					^51^
SA Adult (cm2)				18,000					^46^
SA Child (cm2)				6600					^46^
CF (L/cm3)				0.001					^51^
IR Adult (L/day)				2.2					^47^
IR Child (L/day)				1.8					^47^
EF (day/year)				350					^42^
ED Adult (year)				70					^46^
ED Child (year)				6					^46^
BW Adult (kg)				70					^52^
BW Child (kg)				15					^52^
AT Adult (day)				25,550					^53^
AT Child (day)				2190					^53^

7 $${HQ}_{dermal/oral}=\frac{{{\text{CDI}}}_{{\text{dermal}}}{/{\text{CDI}}}_{{\text{oral}}}}{{{\text{RfD}}}_{{\text{dermal}}}{/{\text{RfD}}}_{{\text{oral}}}}$$

8 $${RfD}_{dermal}={RfD}_{oral}\times ABS$$

9 $$HI=\sum HQ$$

RfD is the reference dose, and ABS is the digestive coefficient of the gastrointestinal tract (Table 1).

The carcinogenic risk (CR) caused by direct digestion and skin contact can be expressed as follows:

10 $${\text{CR}}=\mathrm{CDI }\times \mathrm{ CSF}$$

where CSF represents the conversion slope factor of HMs (Table 1).

### Monte Carlo simulation

Monte Carlo simulation is a technique used in risk assessment to decrease uncertainty related to heavy metal (HM) concentrations and exposure parameters and predict the carcinogenic and non-carcinogenic risks. By adopting this method, researchers can obtain estimations of health risk values^9,19^. Python software, version 3.9.7, is commonly employed for implementing Monte Carlo simulations. Therefore, in the current study, Python code was running for 10,000 iterations and utilized to conduct Monte Carlo simulations and calculate the probability risks associated with both carcinogenic and non-carcinogenic risks of heavy metals for adults and children.

## Results and discussion

### Physicochemical parameters

The hydrochemistry was evaluated based on the physicochemical parameters and heavy metals (Table 2).

**Table 2:** Statistical properties of the investigated parameters in water resources of Siwa Oasis.

Parameters	Min	Max	Mean	SD
pH	6.8	8.7	7.9	0.3
TDS	1120	153,589	9834.1	20,701.9
K^ + ^	3.5	83	42.8	18.5
Na^ + ^	192	39,500	2240.9	5531.6
Mg^2 + ^	9	12,216.6	676.6	1388.8
Ca^2 + ^	19.6	2508.8	366.5	401
Cl^−^	580	94,250	5933.9	13,042.3
SO_4_^2−^	5	5348.7	486.6	652.4
HCO^3−^	83.7	328.8	166.7	36.5
CO_3_^2−^	0	35.3	6.2	8.8
Cd	0.002	0.19	0.04	0.03
Cr	0.0015	12.3	0.6	1.63
Cu	0.002	15.6	1.14	3.004
Fe	0.003	36.2	2.16	5.35
Mn	0.0002	3.37	0.28	0.68
Ni	0.0001	0.72	0.1	0.12
Pb	0.002	2.23	0.33	0.34
Zn	0.0002	0.1	0.03	0.024

The units of all chemical parameters were measured in mg/L except pH.

The total dissolved solids (TDS) values in the studied water samples ranged from 1120 mg/L in the TCA to 153,589 mg/L in the salt lakes, with an average value of 9834.1 mg/L. The pH values of the samples fell within the range of 6.8 to 8.7, indicating neutral to alkaline water conditions. Calcium concentrations ranged from 19.6 mg/L to 2508.8 mg/L, while magnesium (Mg^2+^) concentrations varied from 9 mg/L in the groundwater to 12,216.6 mg/L in surface lakes. Potassium (K^+^) ranged from a minimum of 3.5 mg/L to a maximum of 83 mg/L, and sodium concentrations spanned from 192 mg/L to 39,500 mg/L. Chloride (Cl^−^) concentrations ranged from 580 mg/L to 94,250 mg/L, while sulfate (SO_4_^2−^) concentrations varied from 5 mg/L to 5348.7 mg/L. Bicarbonate (HCO_3_^−^) concentrations ranged from 83.7 mg/L to 328.8 mg/L. The water resources in Siwa Oasis were classified as follows: TCA, springs, and drains were categorized as brackish to saline water, while the surface lakes were classified as hypersaline based on their TDS values. According to WHO^50^, most of the physicochemical parameters in the majority of water samples exceeded the standard limits, rendering them unsuitable for drinking purposes. According to FAO standards^54^, TDS, Ca^2+^, Mg^2+^, Na^+^, K^+^, Cl^-^, and SO_4_^2-^ exceeded the limits for irrigation water in 92%, 24%, 99.2%, 42.1%, 2.2%, 91.7%, and 5.3% of the water samples, respectively. The high concentrations of Cl^-^ and Mg^2+^ have the potential to increase soil salinity and reduce plant production, necessitating further treatment of irrigation water.

The average concentrations of heavy metals in the water samples were as follows: Cd (0.04 mg/L), Cr (0.6 mg/L), Cu (1.14 mg/L), Fe (2.16 mg/L), Mn (0.28 mg/L), Ni (0.1 mg/L), Pb (0.33 mg/L), and Zn (0.03 mg/L). These concentrations are ranked in descending order as follows: Fe > Cu > Cr > Pb > Mn > Ni > Cd > Zn. Notably, the mean concentrations of Fe, Cd, Cr, Pb, and Mn exceeded the standard limits set by WHO^50^, while the concentrations of the other heavy metals remained within the limits.

### Surface water and groundwater origin

**Figure 3:**
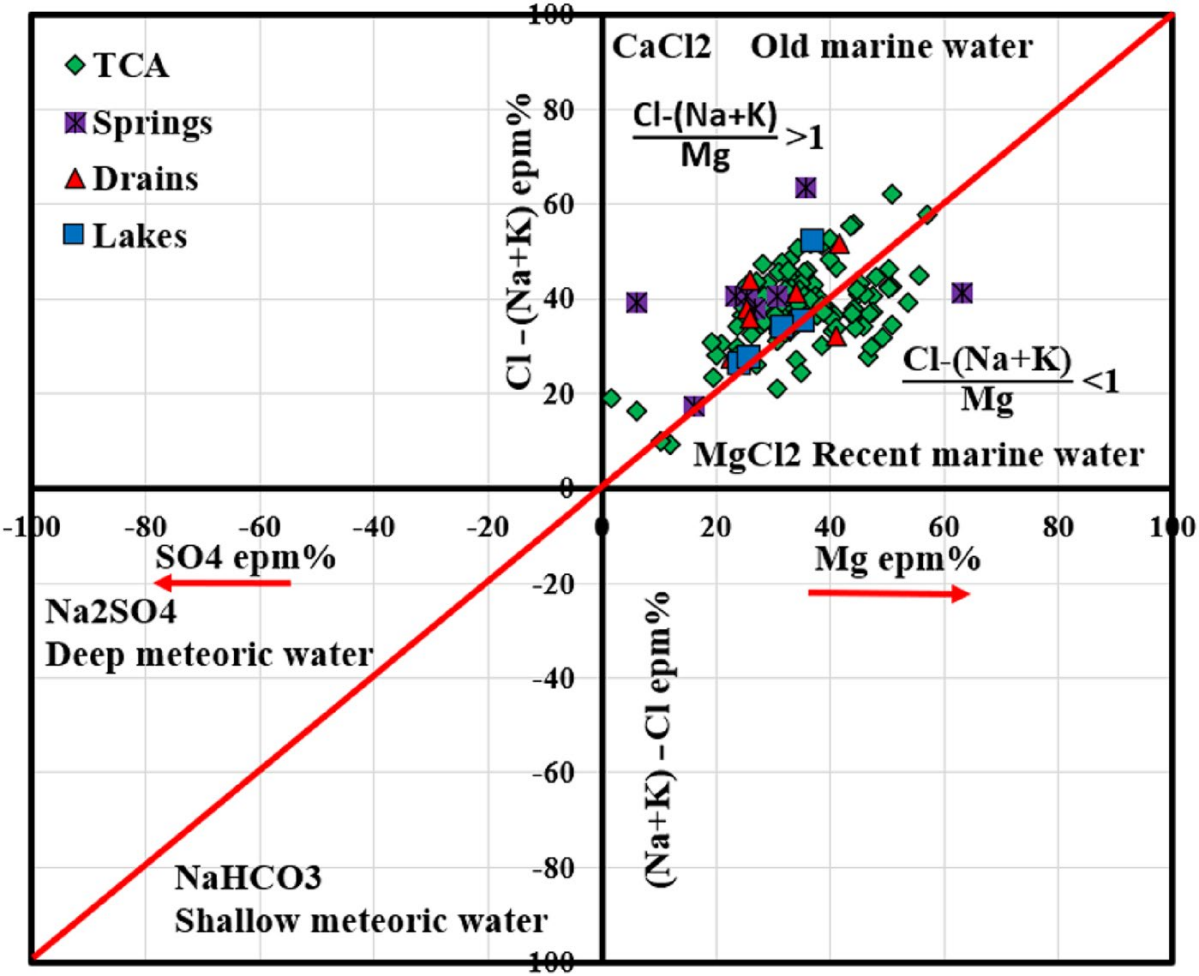
Sulin graph showing the origin and type of water samples in Siwa Oasis.

Figure 3 illustrates the origin of surface water and groundwater samples in Siwa Oasis using the Sulin graph^55^. Firstly, a significant amount (31.5%) of the water samples in the TCA is located in an area associated with recent marine water origin and has a composition rich in MgCl_2_. On the other hand, most of the TCA water samples, water from springs, drains, and salt lakes, fall into the category of old marine water origin characterized by a CaCl_2_ composition.

The higher levels of sodium (Na^+^) and potassium (K^+^) in these samples indicate they may have come from meteoric water through upward flow from the deep Nubian sandstone aquifer (NSSA). However, it is important to note that all water samples share a common marine origin due to the geological makeup of the Tertiary carbonate aquifer. This aquifer predominantly consists of deposits like limestone and dolomite. The presence of these marine deposits (dolomite and limestone) indicates the old presence of seawater trapped within the aquifer system. The high salt content in the groundwater of the TCA area can be attributed to factors including the influence of marine activities and the dissolution of minerals like calcite and dolomite found in the geological formations. Despite this marine influence, the primary source of water supply to the TCA appears to be the upward flow from the deep NSSA with minimal contributions from rainfall in the arid Siwa Oasis region^56^. Furthermore, the contribution of freshwater from NSSA through fault planes does not significantly impact the origin of the TCA samples, as indicated in Fig. 3.

### Geochemical Processes controlling water chemistry

**Figure 4:**
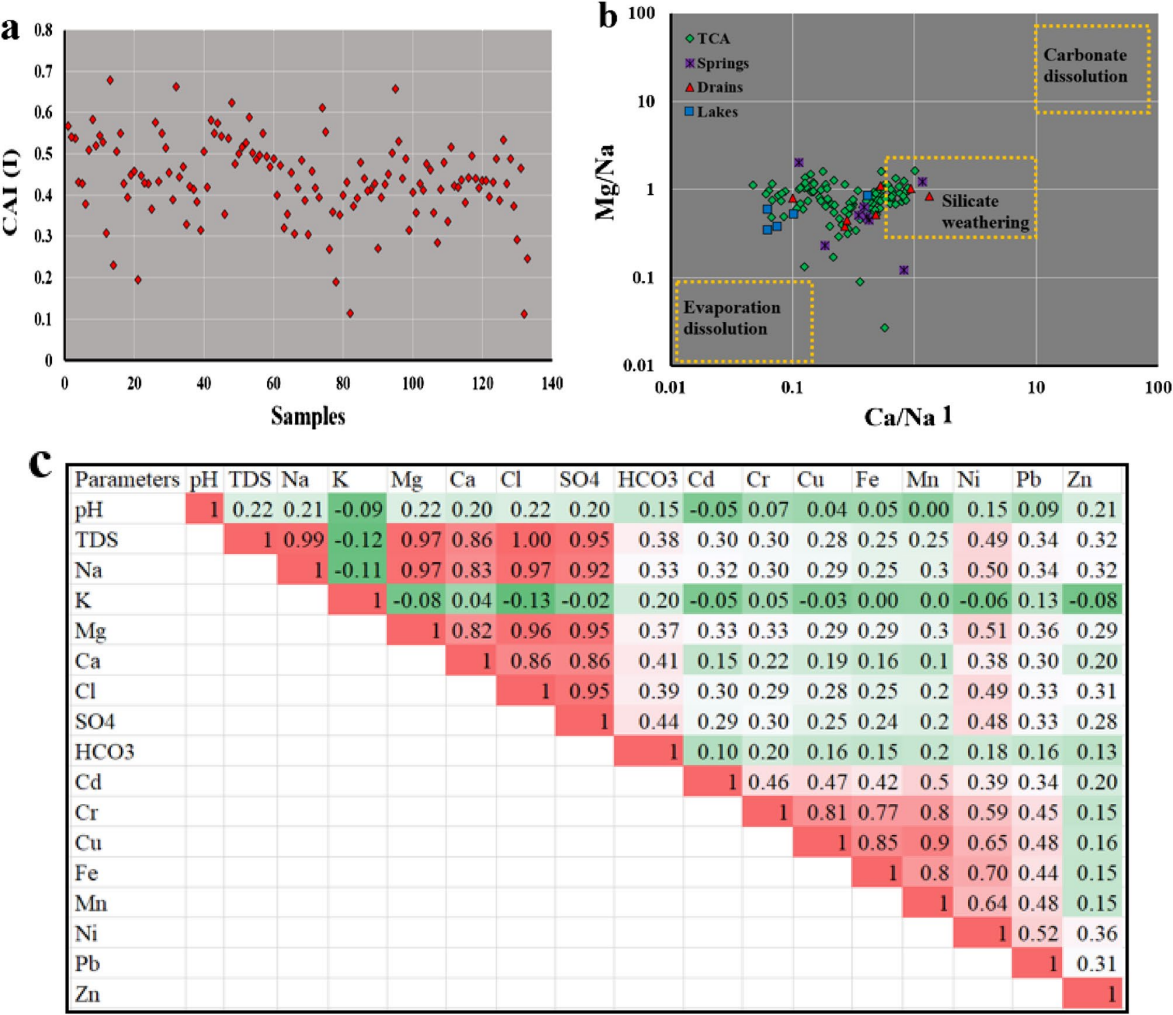
Chloro alkaline index (**a**), bivariate plot of Ca^2+^/Na^+^ versus Mg^2+^/Na^+^ (**b**), and Pearson correlation matrix (**c**).

The presence of clay minerals in the system can have an impact on the mineralization of groundwater by facilitating ion exchange processes. Clay minerals tend to balance their charge by adsorbing monovalent cations like Na^+^ and K^+^ while releasing Ca^2+^ and Mg^2+^ or vice versa. The Chloro alkaline index (CAI) serves as a tool for identifying ion exchange mechanisms between minerals in the aquifer and groundwater. A positive CAI-I value indicates a reverse ion exchange process, whereas a negative value suggests that ion exchange processes control the chemistry of water^57,58^. In this study, all samples showed positive CAI-I values (Fig. 4a), indicating reverse ion exchanges between K^+^ and Na^+^ ions in water and Mg^2+^ and Ca^2+^ ions in the surrounding rock.

To gain insight into the mechanism and weathering type controlling the water chemistry, bivariate plots were used considering the ratio of Ca^2+^/Na^+^ versus Mg^2+^/Na^+^. These plots (Fig. 4b) revealed that silicate weathering plays a significant role in surface water and groundwater composition in Siwa Oasis. However, specific samples fell within zones associated with evaporate dissolution. The study area is mainly characterized by limestone and dolomite in the TCA. These formations receive water from the Nubian aquifer, which comprises sandstone with shale and clay layers. It is worth noting that there are shale and clay layers present between the TCA and NSSA, indicating that alumina silicates might be involved in silicate weathering processes.

Additionally, Pearson's correlation matrix of inter-elemental relationships provides valuable insights into the sources and routes of heavy metals and major ions. This analysis helps elucidate how various heavy metals are linked and provides information about their origins in the groundwater system. In the water samples collected from the Siwa area, it showed a very significant correlation of TDS–Na (r = 0.99), TDS–Cl (r = 1), TDS–Mg (r = 0.97), TDS–Ca (r = 0.86), TDS–SO_4_ (r = 0.95), Na–Mg (r = 0.97), Na–Ca (r = 0.83), Na–Cl (r = 0.97), Na–SO_4_ (r = 0.92), Mg–Cl (r = 0.96), Mg–SO_4_ (r = 0.95), Mg–Ca (r = 0.82), Mg–SO_4_ (r = 0.86), Cr–Cu (r = 0.81), Cr–Fe (r = 0.77), Cr–Ni (r = 0.59), Cr–Mn (r = 0.8), Cu–Fe (r = 0.85), Cu–Mn (r = 0.9), Cu–Ni (r = 0.65), Mn–Ni (r = 0.64), and Pb–Ni (r = 0.52) as shown in (Fig. 4c). The analysis of the relationship between Total Dissolved Solids (TDS) and major ions in both surface water and groundwater provides insights into the factors that contribute to increased salinity in the study areas' water resources. Correlations among ions were observed, indicating the presence of specific minerals and processes that affect water salinity.

The strong correlation between sodium and chloride ions suggests the existence of halite salt in the aquifer system. This indicates that as it dissolves into groundwater, it can lead to increasing water salinity. The correlation between calcium and magnesium suggests the presence of carbonate minerals such as dolomite in the aquifer system. Dolomite dissolution can contribute to elevated Ca and Mg ion levels in water. Similarly, the correlation between calcium (Ca^2+^) and sulfate (SO_4_^2−^) indicates the presence of gypsum minerals in the TCA. Gypsum dissolution can increase calcium and sulfate concentrations in water, thereby contributing to salinity levels. According to heavy metals, the analysis reveals a contribution from various human activities in the study area. These activities include agriculture practices, improper sanitation methods, and discharge from sources as organic decomposition. These activities carried out by humans result in the release of heavy metals into the water resources of Siwa Oasis.

### Cluster analysis of physicochemical parameters and heavy metals

**Figure 5:**
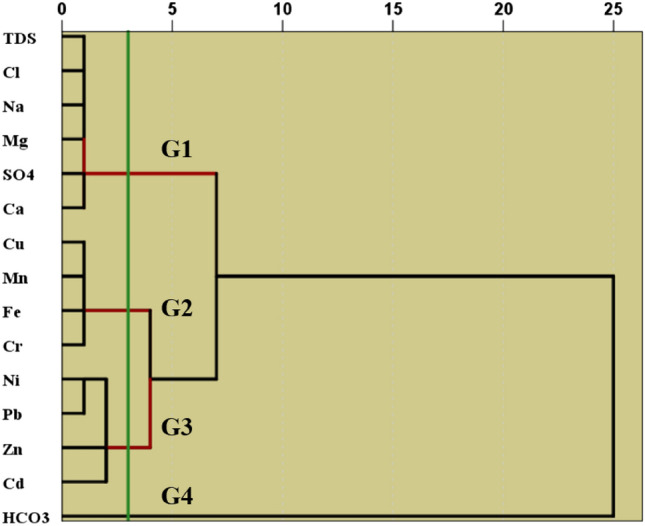
Cluster analysis of the investigated parameters in water samples using dendrogram.

The analysis of groundwater samples using a combination of the Wards linkage method and Euclidean distance revealed three groups (Fig. 5) based on their chemical characteristics;

Group 1 (G1): This group consisted of parameters such as total dissolved solids (TDS), sodium (Na^+^), calcium (Ca^2+^), magnesium (Mg^2+^), sulfate (SO_4_^2−^), and chloride (Cl^−^). These parameters are related to carbonates and evaporite components. The strong correlation between sulfate and chloride indicates that chlorides and salts significantly contribute to groundwater salinity in this region. Moreover, the dominance of Mg^2+^ and Ca^2+^ suggests a connection between carbonate properties and the mineralization process in groundwater.

Group 2 (G2): This group included metals like copper (Cu), chromium (Cr), iron (Fe), and manganese (Mn). These metals may have a common source and be influenced by similar redox environments potentially associated with varying geological compositions at different depths.

Group 3: G3 comprised the remaining metals, nickel (Ni), zinc (Zn), lead (Pb), and cadmium (Cd). Similar to G2, G3 suggests that these heavy metals can exist in groundwater through sources possibly influenced by redox conditions based on geological factors.

Group 4: G4 was identified by bicarbonate (HCO_3_^−^) ions. Unlike G1, which was linked to the dissolution of carbonate minerals, G4 suggests that the bicarbonate found in the groundwater comes from a source possibly resulting from processes like silicate weathering. In essence, this analysis of clusters provides insights into the characteristics and origins of various components present in the water resources of the study areas. It highlights how factors such as mineralization processes and the existence of heavy metals influence the quality of water.

### Principal component analysis (PCA)

**Figure 6:**
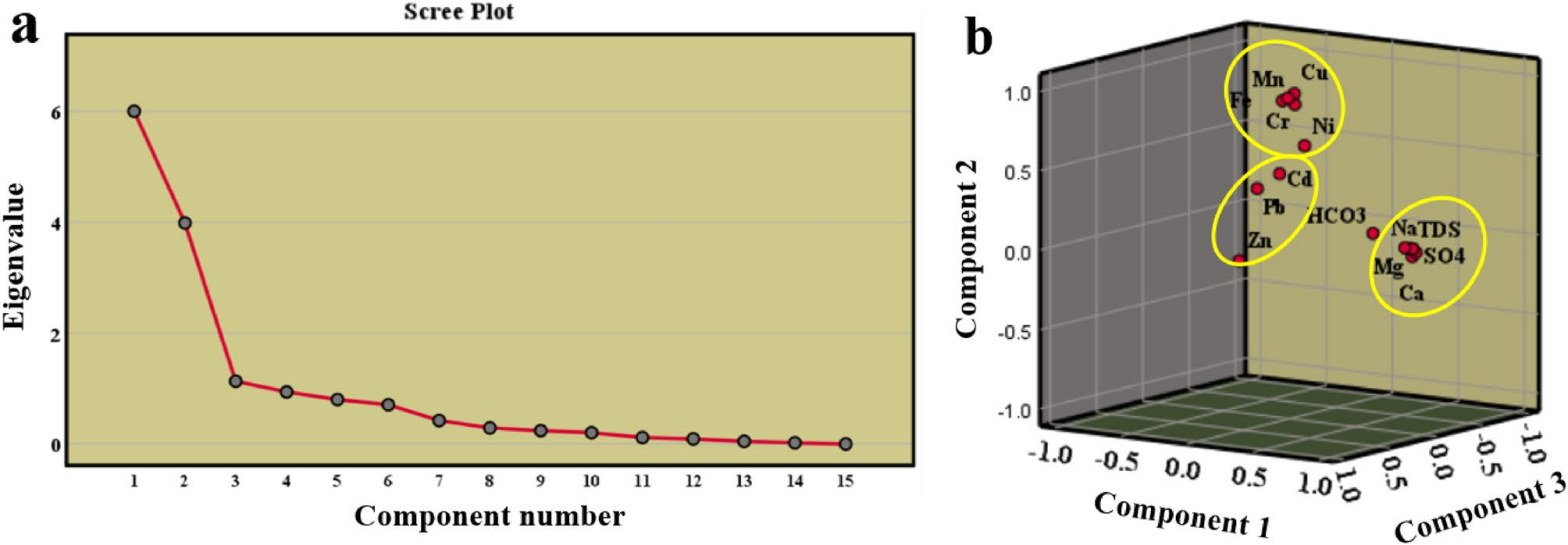
Principal components extracted from scree plot (**a**) and its visualization on 3D plot (**b**).

The principal component analysis (PCA) was conducted to reduce the dimensionality of the dataset and identify underlying patterns in the water chemistry data. To determine if PCA could be applied, the Kaiser Meyer Olkin (KMO) was observed^35^, which resulted in a value of 0.6. This value is higher than 0.5. Additionally, Bartlett's test of sphericity showed a result (0.000, less than 0.05), indicating that the data was suitable for PCA. After conducting PCA, three components (PC1, PC2, and PC3) were extracted from a scree plot with an eigenvalue greater than 1 (Fig. 6a,b). These components explained the proportions of variability in the data; PC1 accounted for 40%, PC2 for 26.5%, and PC3 for 7.5% (Table 3). The variable loadings were examined to understand the strength of the relationship between these components and the original variables used in the analysis. Variables with loadings close to 1 had a strong link with the respective principal component.

**Table 3:** The principal component analysis of the physicochemical parameters and heavy metals in the water samples.

Parameters	PC1	PC2	PC3
TDS	**0.984**	0.068	0.076
Na	**0.958**	0.079	0.122
Mg	**0.962**	0.069	0.069
Ca	**0.884**	−0.01	−0.064
Cl	**0.978**	0.066	0.064
SO4	**0.958**	0.033	0.002
HCO3	0.341	0.031	−0.415
Cd	0.091	0.476	0.238
Cr	−0.02	**0.856**	−0.084
Cu	0.032	**0.94**	−0.004
Fe	−0.063	**0.885**	−0.013
Mn	0.005	**0.914**	0.026
Ni	0.326	0.686	0.304
Pb	0.103	0.421	**0.498**
Zn	0.173	0.015	**0.794**
Eigenvalues	6	3.9	1.1
% of Variance	40	26.5	7.5
Cumulative %	40	66.5	74.1

PC1 (salinization factor): The strong association between PC1 and variables such as Mg^2+^, Ca^2+^, Na^+^, Cl^−^, SO_4_^2−^, and TDS can refer to PC1 as a salinization factor (Table 3). The salinity in the water is likely caused by processes such as the weathering of limestone, halite dissolution, gypsum, and the exchange of ions between groundwater and surrounding rock of TCA. Additionally, human activities like irrigation practices and fertilization may also contribute to the presence of calcium (Ca^2+^), sodium (Na^+^), and magnesium (Mg^2+^), where agriculture is the main activity in Siwa Oasis.

Regarding PC2 (alkaline and contamination factor), there is a relationship between this component and variables such as manganese (Mn), iron (Fe), copper (Cu), nickel (Ni), and chromium (Cr) (Table 3). This suggests that PC2 represents factors related to alkaline conditions and contamination. The interaction between alkaline water, rocks, and soil, oxidation–reduction processes, and potential contamination may contribute to the presence of these metals. The concentrations of iron and manganese can vary depending on whether the groundwater oxidized or reduced. Geogenic processes influence groundwater elements like heavy metals affected by natural processes, including pH and mineral dissolution^2,9^. High levels of these metals could indicate contamination from discharges or natural mineralization processes. According to previous studies^59^, the tertiary carbonate rocks contain glauconite and Fe oxides detrital grains in the northwestern desert of Egypt. Moghra Formation in the study area contains about 1.6–36.1%, 0–0.6%, and 5–50 mg/l of Fe_2_O_3_, MnO, and Cu, respectively. This suggests the geogenic source of these metals in the water resources of Siwa Oasis.

As for PC3 (anthropogenic metal source), it is associated with variables zinc (Zn), cadmium (Cd), and Lead (Pb) (Table 3). Unlike the previous components, these metals were not recorded in the geological formations, suggesting an anthropogenic source for Zn, Cd, and Pb in the studied water.

High concentrations of the investigated heavy metals in irrigation water extracted from TCA can cause serious problems like mineralization and waterlogging through different processes such as ion exchange, cation imbalance, mineral precipitation, sodicity, and salinity. When heavy metals like cadmium, lead, and zinc displace cations on soil exchange sites, it can change the chemistry of the groundwater and the potential supersaturation of minerals. Furthermore, when heavy metals interact with ions in the soil water, it can cause minerals like gypsum to precipitate, reducing soil permeability and worsening waterlogging issues^60^. The formation of hardpans or cemented layers due to the precipitation of iron and aluminum oxides further limits water movement in the soil. Elevated concentrations of heavy metals can negatively impact soil microbial activity. Microorganisms play a crucial role in organic matter decomposition and nutrient cycling^61^. Reduced microbial activity can result in the accumulation of organic matter, further contributing to waterlogging issues.

### Heavy metal pollution index (HPI) and metal index (MI)

The Heavy Metal Pollution Index (HPI) is a tool used for assessing the pollution level of heavy metals in both surface water and groundwater. It helps evaluate the impact of metals on water quality and aids in monitoring and managing health risks associated with exposure to these metals^11^. The HPI values ranged from 111.7 to 7274.5 in the water samples. All the water samples collected were categorized as having high pollution risk and not suitable for drinking according to the HPI classification^38^ (HPI > 100) (Table 4).

**Table 4:** The environmental and health risk indices.

Criteria	Min	Max	Mean	Range	Class	Samples (%)
MI	6.5	462	72.3	MI < 0.3	Very clean	0 (0%)
				0.3 < MI < 1	Clean	0 (0%)
				1 < MI < 2	Partly affected	0 (0%)
				2 < MI < 4	Moderately affected	0 (0%)
				4 < MI < 6	Heavily affected	0 (0%)
				MI > 6	Severely affected	133 (100%)
HPI	111.7	7274.5	1702.9	< 25	Excellent	0 (0%)
				26—50	Good	0 (0%)
				51—75	Poor	0 (0%)
				76—100	Very poor	0 (0%)
				> 100	Unsuitable	133 (100%)
HI Adult (Oral)	1.6	142.1	14.04	< 1	Low risk	0 (0%)
				> 1	High risk	133 (100%)
HI Child (Oral)	6.2	542.6	53.6	< 1	Low risk	0 (0%)
				> 1	High risk	133 (100%)
HI Adult (Dermal)	0.07	47.8	2.6	< 1	Low risk	108 (80.6%)
				> 1	High risk	26 (19.4%)
HI Child (Dermal)	0.2	141	7.7	< 1	Low risk	30 (22.4%)
				> 1	High risk	103 (77.6%)
CRCd Adult (Oral)	0.0003	0.03	0.007	< 1 × 10–4	Acceptable	30 (22.4%)
				> 1 × 10–4	High risk	103 (77.6%)
CRCr Adult (Oral)	2.26E-05	0.18	0.009	< 1 × 10–4	Acceptable	5 (3.7%)
				> 1 × 10–4	High risk	128 (96.3%)
CRPb Adult (Oral)	3.16E-05	0.03	0.005	< 1 × 10–4	Acceptable	2 (1.5%)
				> 1 × 10–4	High risk	131 (98.5%)
CRCd Child (Oral)	0.001	0.1	0.03	< 1 × 10–4	Acceptable	0 (0%)
				> 1 × 10–4	High risk	133 (100%)
CRCr Child (Oral)	8.63E-05	0.7	0.03	< 1 × 10–4	Acceptable	2 (1.5%)
				> 1 × 10–4	High risk	131 (98.5%)
CRPb Child (Oral)	0.0001	0.1	0.02	< 1 × 10–4	Acceptable	0 (0%)
				> 1 × 10–4	High risk	133 (100%)
CRCd Adult (Dermal)	0.002	0.2	0.04	< 1 × 10–4	Acceptable	0 (0%)
				> 1 × 10–4	High risk	133 (100%)
CRCr Adult (Dermal)	0.0002	1.7	0.08	< 1 × 10–4	Acceptable	0 (0%)
				> 1 × 10–4	High risk	133 (100%)
CRPb Adult (Dermal)	1.5E-05	0.01	0.002	< 1 × 10–4	Acceptable	7 (5.3%)
				> 1 × 10–4	High risk	126 (94.7%)
CRCd Child (Dermal)	0.005	0.5	0.1	< 1 × 10–4	Acceptable	0 (0%)
				> 1 × 10–4	High risk	133 (100%)
CRCr Child (Dermal)	0.0006	5.2	0.2	< 1 × 10–4	Acceptable	0 (0%)
				> 1 × 10–4	High risk	133 (100%)
CRPb Child (Dermal)	4.43E-05	0.04	0.007	< 1 × 10–4	Acceptable	3 (2.2%)
				> 1 × 10–4	High risk	130 (97.8%)

**Figure 7:**
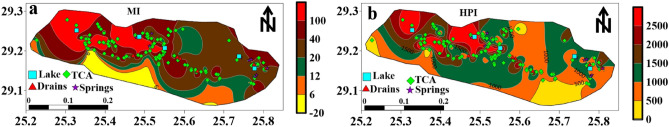
Distripution map of metal index (**a**) and heavy metal pollution index (**b**).

The MI (Metal Index) method was used alongside the HPI index to understand how heavy metals affect water quality. This allowed us to assess the extent of metal contamination in water by comparing it with the maximum allowable limit values outlined in WHO guidelines^48^. The average MI values were between 6.5 and 462 (Table 4). These results indicate high impact and contamination of heavy metals in Siwa Oasis water resources according to MI classification^40^ 38. It highlights the need for monitoring and improving water quality in the OasisOasis. In general, both the HPI and MI evaluations bring attention to the presence of heavy metals in the water resources, which could threaten the environment and humans in Siwa Oasis. This underlines the urgency of tackling this pollution and safeguarding the well-being of the people living there and the surrounding environment. The distribution maps of HPI and MI by using the kriging method showed that the most vulnerable area with heavy metals is the central and western part of Siwa Oasis, which could be due to the over-pumping of groundwater for irrigation purposes (Fig. 7a,b).

### Health risk assessment

The non-carcinogenic and carcinogenic risk hazard indices (HI) were assessed by calculating ingestion and dermal absorption pathways' hazard quotients (HQ). The outcomes reveal the combined potential health risks for humans from exposure to different heavy metals for both children and adults.

### Non-carcinogenic health risk

**Figure 8:**
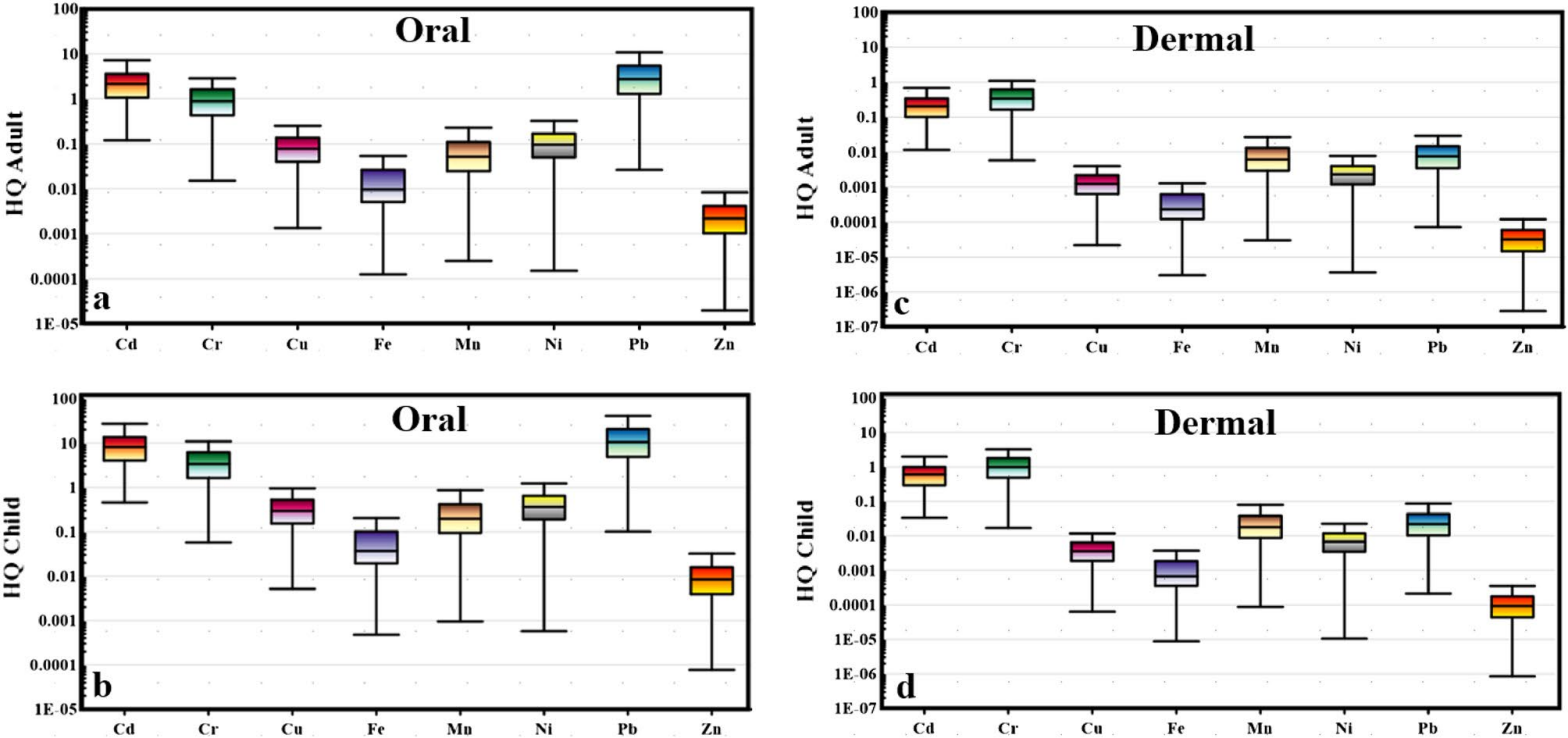
Box plot of the hazard quotient (HQ oral) in adult (**a**), (HQ oral) in child (**b**), (HQ dermal) in adult (**c**), and (HQ dermal) in child.

The toxic elements cadmium (Cd), chromium (Cr), copper (Cu), iron (Fe), manganese (Mn), nickel (Ni), lead (Pb), and zinc (Zn) were evaluated to determine the non-carcinogenic risk in both child and adult. For adults, the hazard quotient (HQ) ingestion ranged from 1.12 to 11.58, 0.015 to 123.8, 0.0013 to 11.7, 0.0001 to 1.5, 0.0002 to 4.2, 0.00015 to 1.08, 0.03 to 28.04 and 2.01E-5 to 0.01 for Cd, Cr, Cu, Fe, Mn, Ni, Pb and Zn, respectively (Fig. 8a). The HQ ingestion for child ranged from 0.46 to 44.2, 0.06 to 472.9, 0.005 to 44.9, 0.0005 to 5.9, 0.001 to 16.17, 0.0006 to 4.1, 0.1 to 107.06 and 0.0001 to 0.04 for Cd, Cr, Cu, Fe, Mn, Ni, Pb and Zn, respectively (Fig. 8b). Based on HQ oral values, the human health risks associated with exposure to Cd, Cr, Cu, Fe, Mn, Ni, Pb, and Zn through ingestion are generally higher for children than adults. It is worth noting that HQ oral values for children and adults are within the permissible limit under 1 for Cu, Fe, Mn, Ni, and Zn. In contrast, the HQ oral value (adult) was more significant than 1 in 76.7%, 45.8%, and 79.7% of the water samples for Cd, Cr, and Pb, respectively. HQ oral value (child) was more significant than 1 in 95.5%, 86.9%, and 94% of the water samples for Cd, Cr, and Pb, respectively. These values are specific to the location and period studied, and the actual human health risks may vary depending on various factors such as exposure duration and frequency, individual susceptibility, and environmental conditions. Nonetheless, the HQ dermal values for adults were in the range of 0.01 to 1.09, 0.005 to 47.02, 2.15E-5 to 0.18, 2.96E-6 to 0.03, 2.98E-5 to 0.5, 3.58E-6 to 0.02, 7.15E-5 to 0.07, and 2.86E-7 to 0.00015 for Cd, Cr, Cu, Fe, Mn, Ni, Pb, and Zn, respectively (Fig. 8c). Moreover, for a child, the HQ dermal values were in the range of 0.03 to 3.24, 0.016 to 138.7, 6.33E-5 to 0.5, 8.74E-6 to 0.1, 8.79E-5 to 1.48, 1.05E-5 to 0.07, 0.0002 to 0.2, and 8.44E-7 to 0.0004 for Cd, Cr, Cu, Fe, Mn, Ni, Pb, and Zn, respectively (Fig. 8d). The HQ dermal values for adults are within the permissible limit under 1 for all heavy metal parameters. In contrast, the HQ dermal value (child) was more significant than 1 in 24% and 50.3% of the water samples for Cd and Cr, respectively, and the rest of the heavy metals fell within acceptable limits. Based on HQ oral and dermal values, the human health risks associated with exposure to Cd, Cr, Cu, Fe, Mn, Ni, Pb, and Zn through dermal exposure are generally higher for children than adults. It is concluded that through oral contact, Cd, Cr, and Pb are the most contributing metals to human health risk (adult and child). In the case of dermal contact, children are more vulnerable to Cd and Cr from the water resources in Siwa Oasis, while there is no risk for adults.

The hazard index (HI) is a valuable indicator used to assess the overall potential health hazard posed by heavy metals in both surface water and groundwater of Siwa Oasis. It considers all possible exposure routes, including ingestion and dermal pathways. The hazard quotients (HQs) associated with each heavy metal and exposure route are summed up to calculate the HI. This comprehensive approach provides a more complete picture of the combined health risks associated with heavy metal contamination in the water resources of Siwa Oasis. The HI value is an essential indicator for assessing the overall health impact and safety of the water sources in the region. HI oral values ranged from 1.6 to 142.1 and 6.2 to 542.6 for adults and children, respectively (Table 4). Moreover, the HI dermal values ranged from 0.07 to 47.8 and 0.2 to 141 for adults and children, respectively (Table 4).

**Figure 9:**
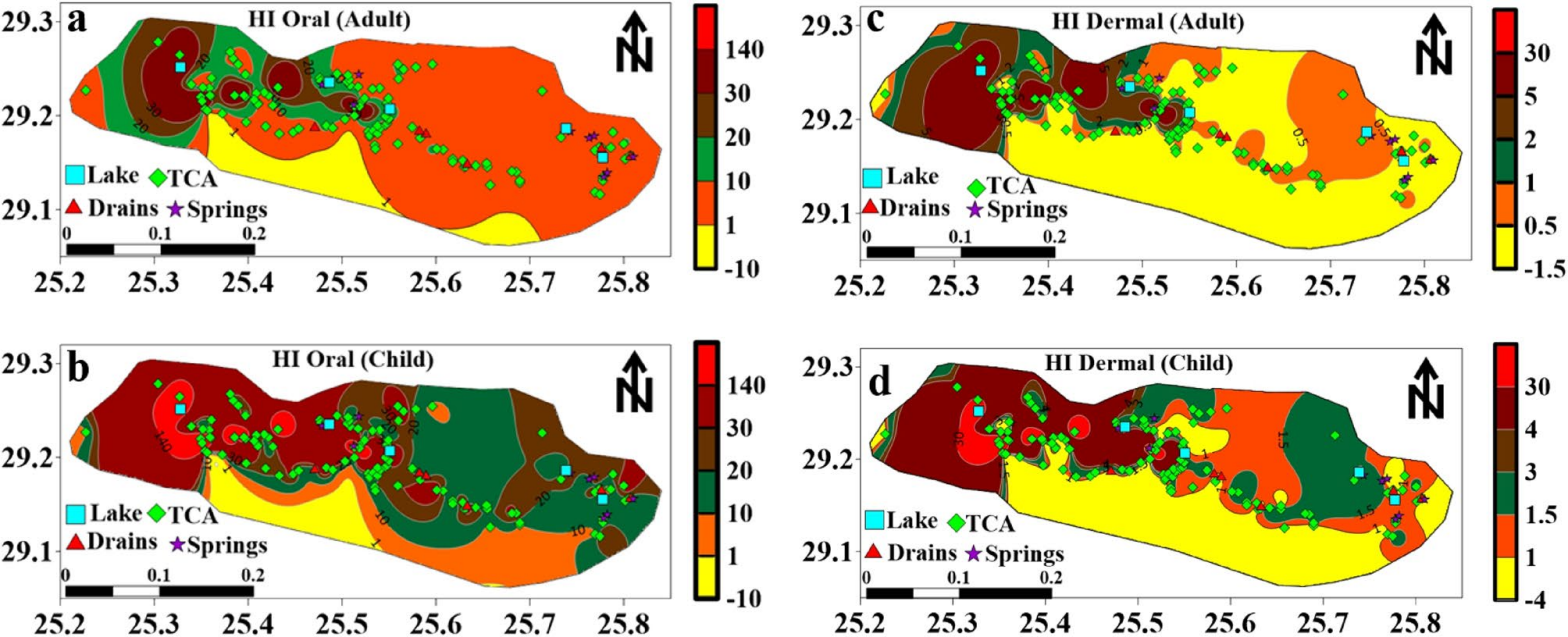
Distripution maps of Hazard index ina adult and child through oral and dermal contact.

It can be concluded that the HI oral values for adults and children were above safe levels (HI > 1) in 100% of the water samples and fell in the high-risk category of non-carcinogenic impact. HI value for adults showed that 80.6% of the water samples fell in the low-risk class, and 19.4% showed a high risk of dermal contact. HI, value for child indicated that 22.4% of the water samples fell in the low-risk class, and 77.6% of the samples showed high risk of dermal contact (Table 4). The result of the HI showed that the child is more vulnerable to oral and dermal contact with heavy metals than adults. However, it is essential to monitor the levels of these metals in the different water resources of Siwa Oasis and their potential health effects, where the groundwater in the study area is non-rechargeable and is the primary water resource for different uses. The distribution maps of the hazard index (HI) in adults and children through dermal and oral contact showed that the central and western parts of Siwa Oasis are the most vulnerable locations to the non-carcinogenic risk impact of heavy metals (Fig. 9).

### Carcinogenic health risk (CR)

**Figure 10:**
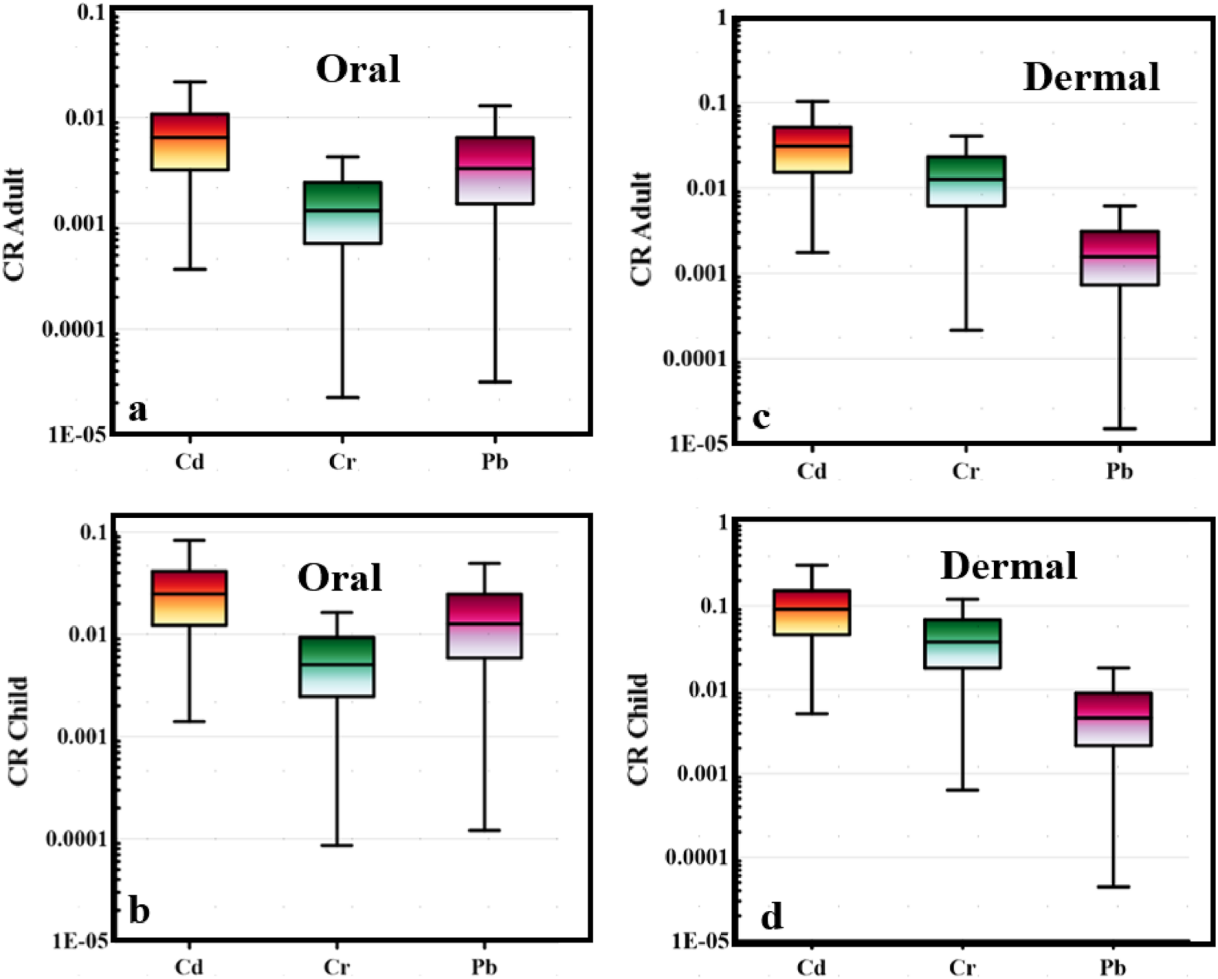
Box plot of the carcinogenic risk (CR) in adult and child through oral (**a** and **b**) and dermal (c and d) contact.

Carcinogenic risks assess the probability of developing cancer as a result of prolonged exposure to a pollutant or a combination of contaminants. The traditional calculation of CR was conducted to calibrate and compare its values with the predicted CR gained from the Monte Carlo simulation later. In the case of adults, the CR oral values fell in a range between 0.0003 and 0.03, 2.26E-05 and 0.18, 3.16E-05 and 0.03 for Cd, Cr, and Pb, respectively (Fig. 10a), while for a child, CR oral values were between 0.001 and 0.1, 8.63E-05 and 0.7, 0.0001 and 0.1 for Cd, Cr, and Pb respectively (Fig. 10b).

Regarding oral contact with heavy metals from the water resources of Siwa Oasis, the high carcinogenic risk (CR > 1 × 10^–4^) for adults was found in 77.6%, 96.3%, and 98.5% of the water samples for Cd, Cr, and Pb respectively and for a child in 100%, 98.5%, and 100 of water samples for Cd, Cr, and Pb respectively (Table 4). On the other hand, for adults, the CR dermal values fell in a range between 0.002 and 0.2, 0.0002 and 1.7, 1.5E-05 and 0.01 for Cd, Cr, and Pb, respectively (Fig. 10c), while for child, CR dermal values were between 0.005 and 0.5, 0.0006 and 5.2, 4.43E-05 and 0.04 for Cd, Cr, and Pb respectively (Fig. 10d). Based on dermal contact with heavy metals from the water samples, the high carcinogenic risk (CR > 1 × 10^–4^) for adults was found in 100%, 100%, and 94.7% of the water samples for Cd, Cr, and Pb respectively and for a child in 100%, 100%, and 97.8% of water samples for Cd, Cr, and Pb respectively (Table 4). The current findings indicated that further treatment is required for all water resources in Siwa Oasis, where the carcinogenic risk from heavy metals is very high and threatens the human health of both children and adults.

### Monte Carlo simulation approach

The Monte Carlo simulation was applied to predict the values of HQ (oral and dermal) of Cd, Cr, Cu, Fe, Mn, Ni, Pb, and Zn, as well as CR (oral and dermal) of Cd, Cr, and Pb for both adults and children.

### Non-carcinogenic health risk

**Figure 11:**
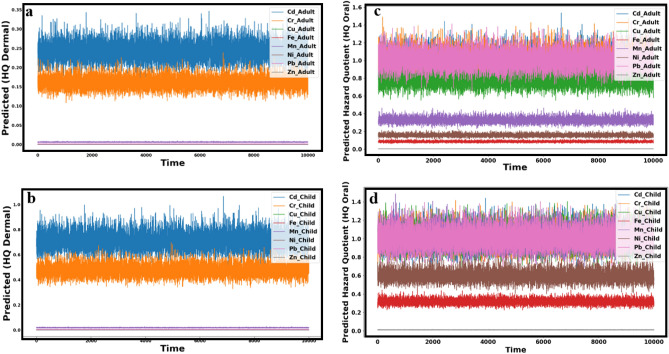
The predicted hazard quotient (HQ dermal) in adult (**a**), (HQ dermal) in child (**b**), (HQ oral) in adult (**c**), and (HQ oral) in child.

The findings, from the Monte Carlo simulation offer insights into the health hazards linked to exposure to heavy metals through various routes in Siwa Oasis. It is reassuring to note that according to the estimated dermal hazard quotient (HQ dermal), there are no indications of any metal exceeding limits (Fig. 11a,b). This suggests that the risk of health issues due to skin contact with water resources is unlikely for adults and children. However, when oral exposure routes are considered, the situation changes. While some heavy metals like Cu, Fe, Mn, Ni, and Zn have predicted HQ values within limits (low risk) for adults, Cd, Cr, and Pb showed estimated HQ values higher than 1 (high risk). This implies a health risk for adults consuming water contaminated with Cd, Cr, and Pb through ingestion. Similar patterns are also observed for children (Fig. 11d,d).

While some heavy metals like Fe, Ni and Zn do not pose risks through oral exposure routes in children, Cd, Cr, Cu, Mn, and Pb show predicted HQ values greater than 1, indicating potential health risks associated with consuming water containing these metals. It is important to remember that these assessments consider assumptions and uncertainties stemming from data sources. Hence, it is crucial to monitor the levels of exposure and regularly update risk assessments to safeguard the water resources in the area and protect the population's health. Through the comparison between the calculated HQ (Fig. 8) and predicted HQ (Fig. 11) through oral and dermal contact with heavy metals, it was found that Cd, Cr, and Pb are the main parameters responsible for high non-carcinogenic impact for child and adult in Siwa Oasis. Monte Carlo simulation was an effective method to predict the HQ successfully.

### Carcinogenic health risk through oral contact

**Figure 12:**
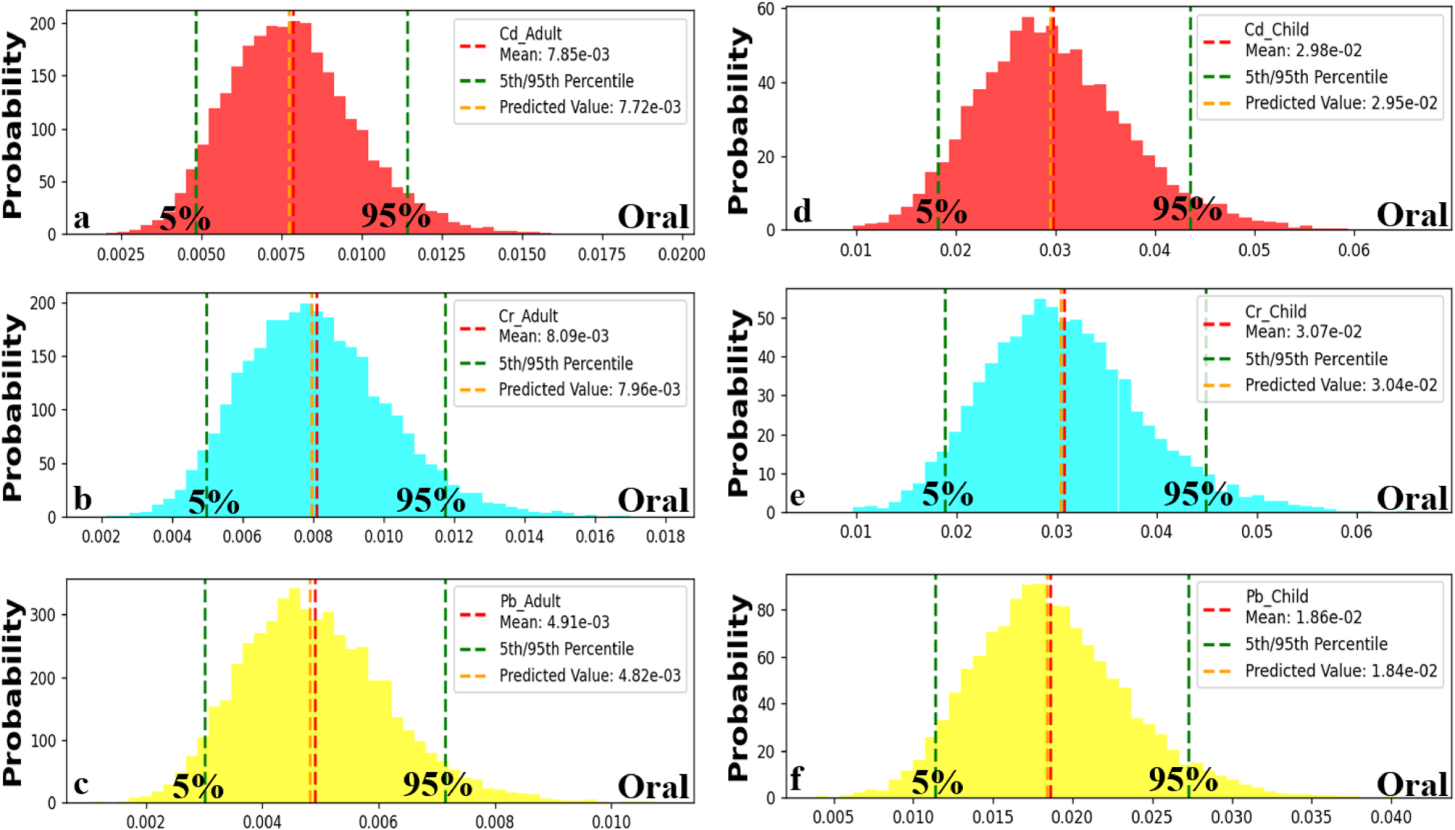
Predicted carcinogenic risk (CR) in adult (**a**, **b** and **c**) and child (**d**, **e** and **f**) through oral contact for Cd, Cr, and Pb respectively.

The analysis of carcinogenic risk probabilities (CR) for oral measurements in children and adults reveals some critical patterns. Across all parameters (Cd et al.), the CR oral measurements are consistently higher in children than adults. For children, the 5th percentile CR oral values (the lower bounds of the estimated cancer risk) were 0.017, 0.019, and 0.012 for Cd, Cr, and Pb, respectively (Fig. 12a–c). On the other hand, at the percentile level 95th (the upper bounds of estimated risk), CR oral values were determined as 0.044, 0.045, and 0.0275 for Cd, Cr, and Pb, respectively (Fig. 12a–c) representing higher potential risks for children. In contrast to this pattern observed in children’s data, the estimated cancer risk levels were relatively lower in adults based on their percentiles. For adults, the lower bounds of estimated cancer risks (5th percentile CR oral values) stood at 0.0047, 0.005, and 0.003 for Cd, Cr, and Pb, respectively (Fig. 12d–f). Furthermore, it was found that at the 95th percentile range, the estimated CR were 0.011, 0.0118, and 0.0072 for Cd, Cr, and Pb, respectively (Fig. 12d–f), suggesting lesser potential risks compared to those observed in children. However, the predicted CR through oral contact showed that most water samples collected from Siwa Oasis have the probability of causing high risk for children and adults with (CR > 1 × 10^−4^).

Through the comparison between the calculated CR (Fig. 8) and predicted CR (Fig. 11) through oral contact with heavy metals, it was found that the three metals (Cd et al.) would have a high carcinogenic impact on children and adults in all water resources of Siwa Oasis. Monte Carlo simulation was an effective method to predict the CR oral successfully.

### Carcinogenic health risk through dermal contact

**Figure 13:**
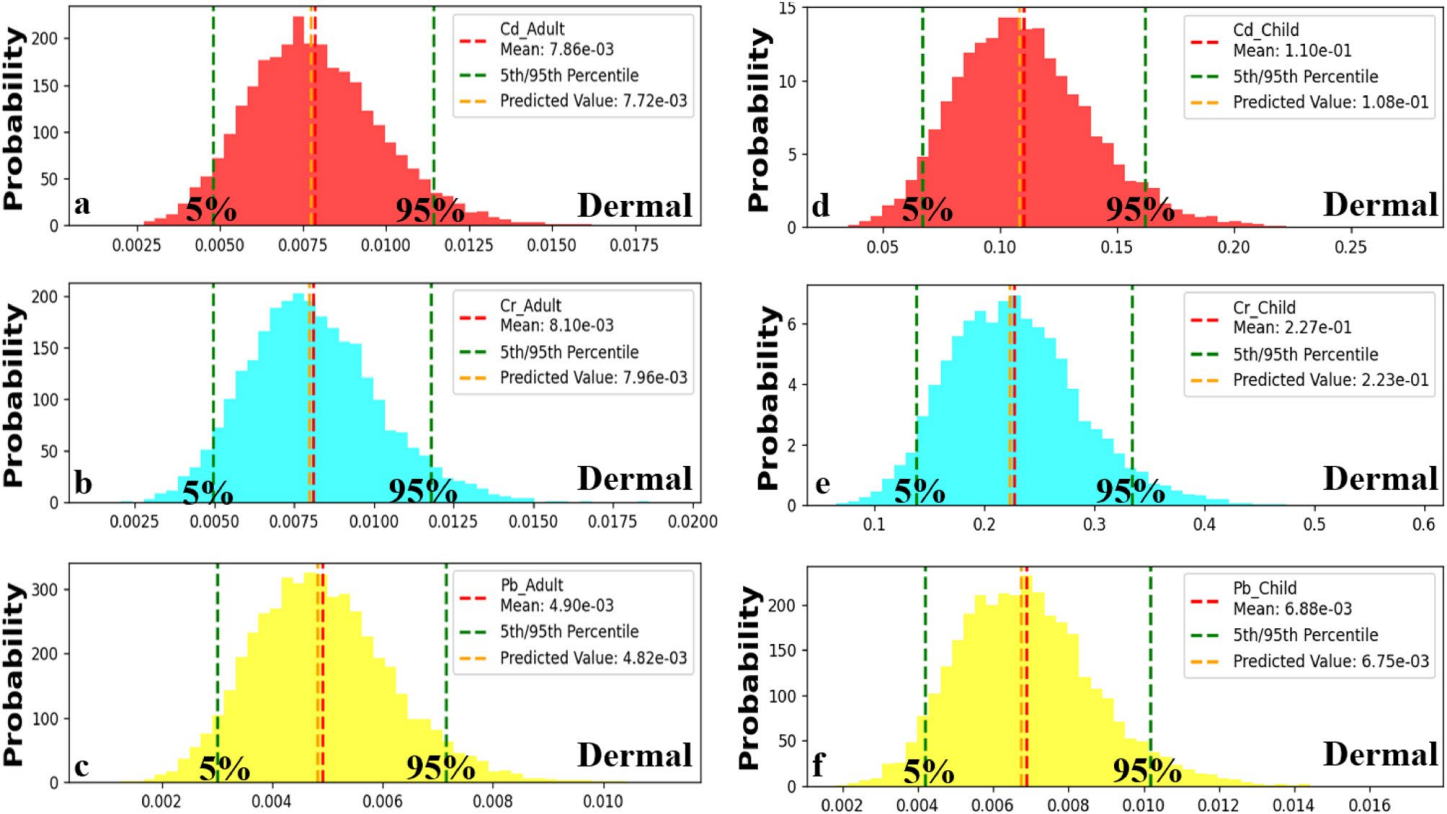
Predicted carcinogenic risk (CR) in adult (**a**, **b** and **c**) and child (**d**, **e** and **f**) through dermal contact for Cd, Cr, and Pb respectively.

The analysis of carcinogenic risk probabilities (CR) due to skin contact in children and adults reveals that children consistently have higher CR values than adults for all parameters (Cd et al.). Regarding children, the estimated 5th percentile CR levels for developing cancer through skin contact were 0.071, 0.13, and 0.0041 for Cd, Cr, and Pb, respectively (Fig. 13a–c). On the other hand, the estimated 95th percentile CR risk levels for developing cancer through skin contact in children were 0.162, 0.33, and 0.0102 for Cd, Cr, and Pb, respectively (Fig. 13a–c). These values indicate the upper boundaries of potential risks from dermal exposure in children. In contrast to children’s results, adults had estimated CR levels for developing cancer through skin contact with values of 0.022, 0.047, and 0.0013 as their lowest percentile (5th) for Cd, Cr, and Pb, respectively (Fig. 12d–f). The 95th percentile CR dermal values for adults were 0.055, 0.112, and 0.0034 for Cd, Cr, and Pb, respectively (Fig. 12d–f), representing the upper bounds of the estimated cancer risk from adult dermal exposure. Overall, the findings indicate that both children and adults are exposed to a high risk of developing cancer due to exposure to Cd, Cr, and Pb found in water resources within Siwa Oasis. The predicted cancer risk levels from the Monte Carlo simulation exceed the acceptable risk level (CR > 1.0E-04) in the majority of water samples, which suggests that continuous exposure to these metals could potentially lead to the development of cancer in the future for both adults and children. These findings emphasize the need to minimize metal contamination in water sources, aiming to reduce carcinogenic health.

Through the comparison between the calculated CR (Fig. 10) and predicted CR (Fig. 13) through dermal contact with heavy metals, it was found that the three metals (Cd et al.) would have a high carcinogenic impact on children and adults in the majority of water samples collected from Siwa Oasis. Monte Carlo simulation was an effective tool for predicting the CR dermal successfully.

This research evaluates the contamination caused by metals in Siwa Oasis. The Heavy Metal Pollution Index (HPI) and Metal Index (MI) reveal surface water and groundwater pollution levels. The HPI values, ranging from 111.7 to 7274.5, classify all water samples as polluted, rendering them unsuitable for drinking. The MI method also emphasizes the impact and contamination of metals, with average MI values ranging from 6.5 to 462. Maps showing distribution patterns highlight western areas of Siwa Oasis as vulnerable potentially due to excessive groundwater pumping for irrigation purposes. Additionally, an analysis based on components reveals both human-related sources of heavy metal pollution. HQ and HI were calculated to fully understand the impact of the metals detected in Siwa Oasis on human health.

The Hazard Quotient (HQ) values for cadmium (Cd), chromium (Cr), and lead (Pb) indicated non-carcinogenic risks to human health. Among these metals, Cd, Cr, and Pb pose risks to children. Long-term exposure to Cd can harm the kidneys and bones, while Cr exposure may cause skin problems. Even low levels of Pb exposure can have cognitive consequences for children. The increased Risk (CR) values for Cd, Cr, and Pb highlight long-term risks. Carcinogenic risk assessments and Monte Carlo simulations further emphasize the urgency for water treatment to mitigate long-term health consequences. These findings collectively emphasize the need for measures to tackle heavy metal pollution and ensure the well-being of the Siwa Oasis community, showcasing the valuable insights provided by this study. However, it is essential to note the limitations of this study, such as its specificity to a location and period, potential variations in health risks, uncertainties associated with data sources, and assumptions made during the Monte Carlo simulation. Despite these limitations, this study emphasizes the need to monitor and manage metal contamination in Siwa Oasis to safeguard the environment and public health. The current study hints at potential ecological consequences, including impacts on soil quality and water resources. The economic repercussions on local agriculture and industries also require attention. The persistence of heavy metals in the environment raises concerns about long-term effects on the ecosystem. Strategies for mitigation and remediation should not only prioritize human health but also aim to preserve the environmental integrity of Siwa Oasis. Regulatory measures and community involvement remain crucial for sustainable solutions. According to the current findings, it is recommended that desalination stations be established to enhance water quality for irrigation in the study area. Additionally, creating companies specializing in salt extraction could provide a dual benefit of addressing heavy metal pollution and utilizing the extracted salts in various industries. By recognizing the broader environmental context and implementing proactive measures, this study emphasizes the urgency of a comprehensive approach to address heavy metal pollution and promote sustainable environmental management in Siwa Oasis. For further research, it would be beneficial to thoroughly understand how heavy metal concentrations vary over time. Conducting studies that cover seasons and years could provide valuable insights into the dynamics of metal pollution. It would also be helpful to investigate water treatment technologies and their effectiveness in reducing levels of metals. Understanding the socio-impacts of metal contamination on communities and industries is crucial for developing holistic management strategies. Lastly, exploring the feasibility and impact of implementing suggested measures like desalination stations and salt extraction companies would provide insights into environmental management in Siwa Oasis.

## Conclusion

This study undertook a comprehensive assessment of heavy metals pollution and associated environmental and health risks in different water resources. The heavy metals included Fe, Mn, Zn, Cu, Ni, Cr, Pb, and Cd. In order to assess risks to both the environment and human health, indices included HPI, MI, HQ, HI, and CR for oral and dermal exposure routes were evaluated. The Monte Carlo method simulates carcinogenic risk assessments, providing a more comprehensive understanding and realistic potential health impacts. In terms of heavy metal concentrations, the mean values ranked as follows: Fe > Cu > Cr > Pb > Mn > Ni > Cd > Zn. Meanwhile, Fe, Cd, Cr, Pb, and Mn exceeded WHO standards. The total dissolved solids (TDS) exhibited significant variability, ranging from 1120 mg/L in the Tertiary Carbonate Aquifer (TCA) to 153,589 mg/L in salt lakes, averaging 9834.1 mg/L. The pH values, ranging from 6.8 to 8.7, indicated neutral to alkaline water conditions. Various ions, including calcium, magnesium, potassium, sodium, chloride, sulfate, and bicarbonate, surpassed recommended limits for irrigation according to FAO standards. The origin of water samples highlights a significant portion of the TCA with recent and old marine water origins. Geochemical processes, including ion exchange facilitated by clay minerals and silicate weathering, contribute to the complex water chemistry. Furthermore, the correlation between heavy metals and various human activities signifies anthropogenic contributions to heavy metal concentrations. The HPI and MI values revealed a risk of pollution across all water resources (HPI > 100 and MI > 6). Moreover, HI oral values were greater than one (HI > 1) in most water samples, indicating high risks associated with the non-carcinogenic effects of these metals on both adults and children. The health risks associated with dermal contact showed a higher risk for children, and 77.6% of water samples have HI > 1. It is still a concern for adults; 19.4% of water samples have an HI value greater than one (HI > 1). Regarding metals like Cd, Cr, and Pb, most water samples indicate that adults and children are vulnerable to carcinogenic effects. The CR values (oral and dermal) for these metals are greater than 1 × 10^−4^ in most samples. The Monte Carlo method further confirmed the presence of carcinogenic impact, with the 5th and 95th percentile risk exposures indicating elevated risks for both children and adults. Additionally, statistical analyses such as cluster analysis and Principal Component Analysis (PCA) provide insights into groundwater composition. The clustering of variables reveals distinct groups based on physicochemical characteristics such as carbonates and evaporites, heavy metals, and bicarbonates, shedding light on the sources and controlling factors of water chemistry. PCA identifies three components where PC1, with 40% of the total variance, represents factors related to limestone weathering and ion exchange processes, which contribute to salinization. PC2, with 26.5 of the total variances, indicates the movement of alkaline water and potential contamination processes particularly associated with heavy metals. PC3, with 7.5 of the total variances, highlights the sources of Zn, Cd, and Pb. Considering these findings, immediate action must be taken to mitigate the dangers of metal pollution in Siwa Oasis. It is crucial to implement treatment strategies for all water resources to protect the environment and human health. Particular attention should be given to preventing carcinogenic and non-carcinogenic health impacts by decreasing exposure duration. This finding showed that the Monte Carlo method is an effective tool that should be applied alongside the traditional calculation of health risk indices to decrease uncertainty and increase the reliability of the results.

## Data Availability

The datasets utilized and/or analyzed during the current study are available upon request from the corresponding author.
